# Peptide receptor radionuclide therapy in neuroendocrine tumours: advances, combination strategies, and future directions

**DOI:** 10.1007/s00259-025-07750-w

**Published:** 2026-02-14

**Authors:** Irene J. Virgolini, Gianpaolo Di Santo, Giulia Santo

**Affiliations:** ¹Department of Nuclear Medicine, Medical University of Innsbruck, Anichstraße 35, Innsbruck, 6020 Austria

**Keywords:** Peptide receptor radionuclide therapy, Neuroendocrine tumour, Targeted alpha therapy, SSTR antagonist, Combination therapy, CCK2 receptor

## Abstract

Peptide receptor radionuclide therapy (PRRT) has established itself as a pivotal component in the management of advanced, somatostatin receptor (SSTR)-positive neuroendocrine tumours (NETs). The NETTER-1 phase III trial demonstrated that [^177^Lu]Lu-DOTATATE significantly prolongs progression-free survival (PFS) and improves quality of life in patients with midgut NETs refractory to somatostatin analogues, leading to regulatory approval by both EMA (2017) and FDA (2018). The recent NETTER-2 phase III trial further extended these findings by supporting the first-line use of PRRT in Grade 2 and 3 gastroentero-pancreatic (GEP)-NETs (Ki-67 ≥ 10 ≤ 55%). Beyond standard β-emitting therapy, several developments are reshaping the field: the clinical adoption of SSTR antagonists such as radiolabelled JR-11 and LM3, targeted α-particle–emitting therapies (^225^Ac, ^212^Pb, ^213^Bi) for resistant disease, and rational combination strategies with chemotherapy, DNA-repair inhibitors, and immunotherapy. Parallel innovation in radiopharmaceutical chemistry has yielded new peptide ligands, including cholecystokinin-2 receptor (CCK2R)–targeted compounds such as DOTA-MGS5, which show promise for rare NETs such as medullary thyroid carcinoma (MTC) and small-cell lung cancer (SCLC). This review summarises clinical evidence, translational advances, and future perspectives for PRRT as a cornerstone of precision nuclear oncology. Emphasis is placed on expanding indications, integrating α-emitters, improving safety and dosimetry, and developing novel theragnostic ligands that enable personalised treatment strategies for NETs patients.

## Introduction

Neuroendocrine tumours (NETs) comprise a heterogeneous group of neoplasms originating from the diffuse neuroendocrine system and characterised by the frequent over-expression of somatostatin receptors (SSTRs), particularly the subtype 2 receptor (SSTR2) [1]. For decades, systemic therapy options for advanced disease were limited to somatostatin analogues, targeted biological agents, or cytotoxic chemotherapy. The development of peptide receptor radionuclide therapy (PRRT) represented a major paradigm shift, enabling selective delivery of therapeutic radiation to tumour cells while sparing normal tissues.

PRRT employs radiolabelled somatostatin analogues—most commonly [^177^Lu]Lu-DOTATATE or [^90^Y]Y-DOTATOC—linked via the chelator DOTA to a peptide backbone with high SSTR2 affinity. Following intravenous administration, the compound binds to SSTR2-expressing cells, is internalised, and deposits β-particle energy locally, leading to DNA damage and apoptosis. First clinical data on the efficacy of [^177^Lu]Lu-DOTATATE were presented by the Rotterdam group [2] in 2008 for gastroentero-pancreatic neuroendocrine tumours (GEP-NETs) and the same group presented their promising results for bronchial NETs in 2017 [3]. The NETTER-1 phase III trial [4] was the first randomised study to demonstrate a clinically significant benefit of PRRT over high-dose octreotide (60 mg) long-acting repeatable (LAR) in patients with midgut NETs progressing on somatostatin analogues. Median progression-free survival (PFS) improved from 8.4 to 28.4 months, and objective response rates rose from 3 to 18%. The final NETTER-1 results presented in 2021 demonstrated a median overall survival (OS) of 11.7 months over high-dose octreotide LAR [5]. Despite lacking statistical significance due to the cross-over design of the study, these results, together with favourable safety data, led to regulatory approval and inclusion into major clinical guidelines such as those of the European Neuroendocrine Tumour Society (ENETS) [6], European Society for Medical Oncology (ESMO) [7], National Comprehensive Cancer Network (NCCN) [8], European Association of Nuclear Medicine/Society of Nuclear Medicine and Molecular Imaging (EANM/SNMMI) [9], and American Society of Clinical Oncology (ASCO) [10]. The optimal patient to be treated with [^177^Lu]Lu-DOTATATE is one with high SSTR expression as assessed by SSTR-positron emission tomography/computed tomography (PET/CT), who has a relatively good Karnofsky performance status score and who is progressive under long-acting somatostatin analogues [11]. Since its approval, PRRT has evolved from a therapy for progressive midgut NETs to a versatile platform for multiple neuroendocrine neoplasms (NENs). Current research directions can be grouped into several complementary strategies:

1. **Extending PRRT to new clinical contexts.**
  The NETTER-2 trial [12] demonstrated that early, first-line application of [^177^Lu]Lu-DOTATATE plus long-acting somatostatin analogues significantly improved PFS by 14 months in Grade 2 and 3 GEP-NETs. Other efforts target rare or aggressive entities, such as meningioma, medullary thyroid carcinoma (MTC), pheochromocytoma and paraganglioma (PPGL), and small-cell lung cancer (SCLC), in which standard therapies are ineffective [13].
2. **Improving tumour targeting and radiation delivery.**
  The transition from SSTR agonists to antagonists has markedly increased tumour-to-background ratios and radiation dose delivery. Novel ligands such as JR-11 (OPS201) and LM3 exhibit enhanced receptor occupancy without internalisation, translating into superior dosimetry and potentially higher efficacy [14].
3. **Introducing targeted α-particle emitters and new isotopes.**
  α-emitting radionuclides (e.g., ^225^Ac, ^212^Pb, ^213^Bi) deliver high-linear energy transfer (LET) radiation over short path lengths, producing irreparable DNA double-strand breaks. Early clinical trials of [^225^Ac]Ac-DOTATATE have reported durable responses even in patients refractory to β-PRRT [15]. New isotopes include emerging ^67^Cu and ^161^Tb programmes.
4. **Designing rational combination strategies.**
  Integrative approaches seek to potentiate PRRT efficacy through radiosensitising agents or immunomodulation [16]. Examples include CAPTEM (i.e., capecitabine + temozolomide) plus PRRT, PRRT with checkpoint inhibitors (e.g., nivolumab, pembrolizumab), and combinations with DNA-repair inhibitors (such as olaparib in the LuPARP trial).
5. **Developing new peptide ligands for rare receptor systems.**
  Expanding beyond SSTRs, novel radiopharmaceuticals target receptors such as cholecystokinin-2 receptor (CCK2R), gastrin-releasing peptide receptor (GRPR), and glucagon-like peptide-1 receptor (GLP-1R). The CCK2R ligand DOTA-MGS5, pioneered in Innsbruck, shows promising preclinical and first-in-human data in MTC and SCLC [17, 18].

Together, these developments are transforming PRRT from a single therapeutic concept into a dynamic and personalised platform for molecular oncology. The subsequent review of each of these domains—clinical expansion, mechanistic optimisation, emerging α-particle therapies, combination regimens, and novel ligand design—culminates in a discussion of future perspectives for integrating PRRT into precision cancer medicine.

## Clinical development of PRRT 

**Fig. 1:**
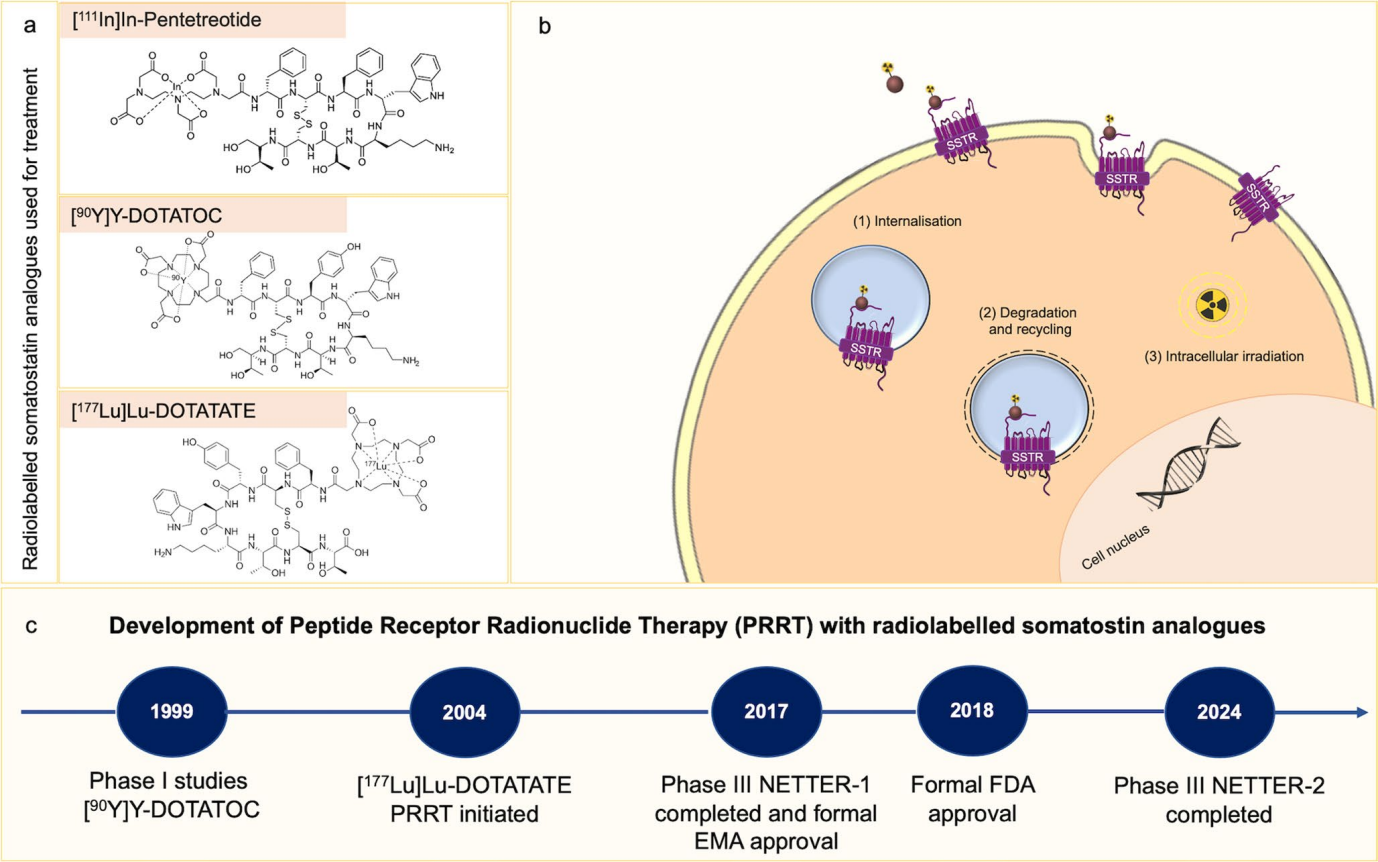
Radiolabelled somatostatin analogues: mechanism of action and key milestones in the development of Peptide Receptor Radionuclide Therapy (PRRT). (**a**) Somatostatin analogues can be labelled with different radioisotopes for therapeutic purposes, such as ^111^In, ^90^Y, or ^177^Lu, with the latter two being the most widely used. (**b**) After binding to somatostatin receptors (SSTRs) expressed by neuroendocrine tumour cells, the radiolabelled compound is internalised, resulting in intracellular irradiation (modified from [26]). (c) Following first clinical applications in the early 90 s [27], phase I studies were initiated, establishing feasibility and dose-limiting renal toxicity [28]. PRRT has progressively evolved through multiple developing stages, leading to its incorporation into clinical practice. Results from the NETTER-2 trial [12] and ongoing clinical studies hold promise for expanding the clinical application of PRRT.

(Figure 1 and Table 1)

**Table 1:** Current clinical trials investigating the extension of PRRT indications to new clinical contexts (status December 2025)

Trial	No Trial	Phase	Patient	*N*	Status
NETTER-2	NCT03972488	III	Metastasized/locally advanced, inoperable well-differentiated G2-G3 (Ki-67 ≥ 10 and ≤ 55%) GEP-NETs diagnosed within 6 months prior to screening	226	Active, not recruiting
NETTER-3	NCT06784752	III	Newly diagnosed well-differentiated G1-G2 (Ki-67 < 10%) advanced GEP-NETs with high disease burden	240	Recruiting
COMPETE	NCT03049189	III	Inoperable, progressive, well-differentiated non-functional GE-NET or both functional or non-functional p-NETs	309	Active, not recruiting
COMPOSE	NCT04919226	III	Unresectable, well-differentiated G2 and G3 GEP-NETs	259	Active, not recruiting
NET RETREAT	NCT05773274	II	Metastatic/unresectable G1-G2 well-differentiated GEP-NETs, including NETs of unknown primary, who have progressed following previous PRRT	100	Recruiting
ReLUTH	NCT04954820	II	Metastatic/unresectable progressive intestinal G1 or G2 NETs previously treated with 4 cycles of PRRT	146	Recruiting
LUMEN-1	NCT06326190	II	Recurrent meningioma (all grades, 1–3 per WHO CNS5)	136	Recruiting
-	NCT05142696	Ib/II	Newly diagnosed Extensive Stage Small Cell Lung Cancer (ES-SCLC)	200	Recruiting

Legend:*GE *gastroenteric, *GEP-NETs* gastroenteropancreatic neuroendocrine tumours, *NETs* neuroendocrine tumours, *p-NET *pancreatic neuroendocrine tumours, *PRRT *peptide receptor radionuclide therapy, *WHO CNS5* fifth edition of the WHO Classification of Tumours of the Central Nervous System

### Evidence from NETTER trials (and beyond)

The clinical establishment of PRRT was founded on the pivotal NETTER-1 trial [4, 5] and further consolidated recently by the phase III NETTER-2 study [12].

NETTER-1 enrolled 229 patients with progressive, well-differentiated midgut NETs and demonstrated a remarkable prolongation of median PFS from 8.4 to 28.4 months with [^177^Lu]Lu-DOTATATE compared to high-dose octreotide LAR. Objective response rates improved from 3% to 18%, and OS analysis confirmed a clinically relevant trend toward longer survival (+ 11.7 months median OS [5]). Toxicity remained modest: grade ≥ 3 haematologic events occurred in < 10% and nephrotoxicity in < 6%. No cases of therapy-related myelodysplastic syndrome (MDS) or acute myeloid leukemia (AML) were recorded during long-term follow-up.

Building upon this foundation, NETTER-2 (NCT03972488) investigated first-line PRRT plus long-acting octreotide LAR in high Grade 2 (Ki-67 ≥ 10–20%) and Grade 3 (Ki-67 ≥ 20–55%) GEP-NETs. Preliminary data revealed a 14-month improvement in PFS compared with long-acting octreotide LAR, with a consistent safety profile (grade ≥ 3 haematologic events occurred in < 14%) [12]. These findings support moving PRRT to earlier lines of therapy and redefining it as a potential disease-modifying modality rather than purely palliative care. The study is active and not recruiting.

The ongoing phase III NETTER-3 (NCT06784752) is designed to evaluate [^177^Lu]Lu-DOTATATE + octreotide LAR versus octreotide LAR alone in newly-diagnosed SSTR-positive G1/G2 GEP-NETs with high disease burden, with primary completion estimated for 2030. At this time, NETTER-3 remains ongoing and no mature results are published yet.

Beyond the NETTER trials, additional clinical settings are being investigated in ongoing studies in NET patients. The COMPETE trial (NCT03049189) is a multicenter phase III study comparing the efficacy and safety of [^177^Lu]Lu-edotreotide PRRT with everolimus in patients with inoperable, progressive, SSTR-positive GEP-NETs. Preliminary results presented at the last ENETS and ESMO congress showed a statistically significant improvement in median PFS with PRRT compared to everolimus (23.9 vs. 14.1 months; HR 0.67; 95% CI 0.48–0.95; stratified *p* = 0.022). [^177^Lu]Lu-edotreotide also achieved higher objective response rates (central ORR 21.9% vs. 4.2%; local ORR 30.5% vs. 8.4%) [19].

Additionally, the phase III COMPOSE trial (NCT04919226) enrolled patients with well-differentiated G2/G3 (Ki-67 15–55%), SSTR-positive GEP-NETs and is currently active but not recruiting. The study evaluates the efficacy, safety, and patient-reported outcomes of first- or second-line [^177^Lu]Lu-edotreotide vs. best standard of care, and results are awaited.

Furthermore, several reports focus on the efficacy of re-challenge PRRT in advanced NET patient settings [20]. Of note, phase 2 randomized clinical trials are now active recruiting to verify the efficacy and safety of re-challenge PRRT (NCT05773274; NCT04954820). Recently, the final results from a multicenter 10-year survival study endorsed by the World Association of Radiopharmaceutical and Molecular Therapy (WARMTH) and the International Atomic Energy Agency (IAEA) [21] showed the prognostic implication of [^18^F]FDG PET/CT for the prediction of PFS and OS in the re-challenge setting and its potential role in guiding treatment decisions in patients who benefited from the initial PRRT.

### Expanding clinical indications

The robust safety profile of [^177^Lu]Lu-DOTATATE has encouraged its compassionate or investigational use in rare and aggressive NENs:

- **Meningioma**:
  Inoperable or recurrent meningioma can be treated with radiosurgery or fractionated radiotherapy. However, once surgical and radiation possibilities have been exhausted, other systemic therapies have shown limited efficacy [22]. Based on the high density of SSTR2 in more than ~ 90% of meningiomas [23], the first randomised clinical trial (NCT06326190) comparing [^177^Lu]Lu-DOTATATE with standard of care has recently begun recruitment, and results are eagerly awaited.
- **Medullary thyroid carcinoma (MTC)**:
  The limited success of kinase inhibitors and the frequent over-expression of SSTRs have stimulated PRRT approaches using somatostatin or gastrin analogues [24]. Compassionate-use cases demonstrate symptomatic improvement and disease stabilisation [13].
- **Pheochromocytoma and paraganglioma (PPGL)**:
  SSTR-targeted PRRT has demonstrated biochemical responses and pain reduction across multiple patient cohorts [13]. Accordingly, its use is supported by major oncological guidelines for unresectable, progressive PPGL [8, 25].
- **Small-cell lung cancer (SCLC)**:
  A phase Ib/II study assessing the safety and efficacy of [^177^Lu]Lu-DOTATATE in newly diagnosed extensive-stage SCLC in combination with carboplatin, etoposide and atezolizumab in induction, and with atezolizumab in maintenance, is currently underway (NCT05142696).

Autoradiography studies show CCK2R expression > 50% and SSTR2 < 30%. Translational experiences with [^68^Ga]Ga-DOTA-MGS5 and therapeutic [^177^Lu]Lu-DOTA-MGS5 suggest feasibility of receptor-mediated targeting in extensive-stage SCLC [18].

These efforts mark a gradual evolution of PRRT from tumour-specific to receptor-driven therapy applicable across neuroendocrine phenotypes.

## SSTR antagonist PRRT

### Rationale

Conventional PRRT uses SSTR agonists (DOTATATE, DOTATOC) that internalise after high-affinity ligand receptor binding. Some evidence suggests that SSTR antagonists bind to more receptor sites per cell and thereby achieve higher tumour uptake despite lacking internalisation [26, 29]. The wider receptor occupancy, slower dissociation, and increased residence time may be the reason to translate into improved tumour-to-organ ratios [30, 31].

### Clinical development

Two leading antagonists are currently under clinical investigation:

- **JR-11 (OPS201;** [^177^ **Lu]Lu-satoreotide-tetraxetan)**:
  Phase I/II studies [32] demonstrated up to 1.8-fold higher tumour uptake than DOTATATE and a 1.5- to 2.5-fold increase in tumour dose while maintaining comparable renal exposure.
- **LM3** ([^**177**^**Lu]Lu-DOTA-LM3)**:
  Early human data indicate favourable biodistribution and potentially higher efficacy at reduced peptide mass [33].

### Dosimetry and safety

Patient-specific dosimetry shows increased tumour absorbed doses (up to 200 Gy) without a proportional rise in kidney or bone marrow dose. Renal dosimetry remains below 0.6 Gy/GBq. Transient grade 1–2 haematologic toxicity and mild nausea are the predominant adverse events [32–34].

### Future directions

Phase II/III comparative trials (e.g., NCT04919226, NCT05477576) are ongoing to evaluate PFS and safety endpoints. Antagonist PRRT is poised to redefine standard PRRT for high-burden or refractory disease.

## Targeted alpha therapy (TAT)

### Concept and rationale

Alpha emitters such as ^225^Ac, ^213^Bi, and ^212^Pb deliver extremely high linear energy transfer (LET) radiation (80–100 keV µm⁻¹) over micrometre distances, causing irreparable DNA double-strand breaks while minimising collateral damage [35]. These features make them ideal for treating micro-metastatic or β-resistant disease [36].

### Clinical experience

Compassionate and prospective studies provide early evidence of efficacy:

- **[**^**225**^**Ac]Ac-DOTATATE:**
  In a prospective initial experience, Ballal et al. [37] in 32 patients with advanced GEP-NETs treated with [^225^Ac]Ac-DOTATATE who had progressed after [^177^Lu]Lu-DOTATATE therapy, achieved an objective response rate of 62.5%. Long-term outcome results from the same group [38] on 91 GEP-NETs patients treated with [^225^Ac]Ac-DOTATATE and capecitabine, showed a 24-month PFS probability of 67.5% and OS probability of 70.8%. Median PFS and OS were not reached. Reported toxicities were predominantly transient haematologic suppression [37, 38].
- **[**^**212**^**Pb]Pb-DOTAMTATE and [**^**212**^**Pb]Pb-VMT-α-NET:**
  Ongoing phase I/II trials (NCT05153772, RayzeBio; NCT03466216, Radiomedix; NCT05636618, Perspective Therapeutics) are exploring safety, dosimetry, and efficacy. Early cohorts show partial responses at low activities [39].
- **[**^**213**^**Bi]Bi-based compounds:**
  Though limited by short half-life, pilot studies confirm feasibility and antitumour activity [40].

### Combination and translational studies

Preclinical data indicate synergy between α-PRRT and DNA-repair inhibitors (e.g., PARPi) or immune checkpoint blockade (anti-PD-1). Proof-of-concept clinical applications suggest enhanced efficacy without additional toxicity [41]. Such findings justify integrated α-PRRT trials combining biological and immune modulation.

### Challenges and outlook

Key limitations include radionuclide supply, daughter isotope recoil, the ability to incorporate them chemically and stably into a suitable vector, and dosimetry standardization [42, 43]. Nonetheless, α-therapy represents the next transformative step toward curative radioligand strategies.

## Combination strategies 

**Fig. 2:**
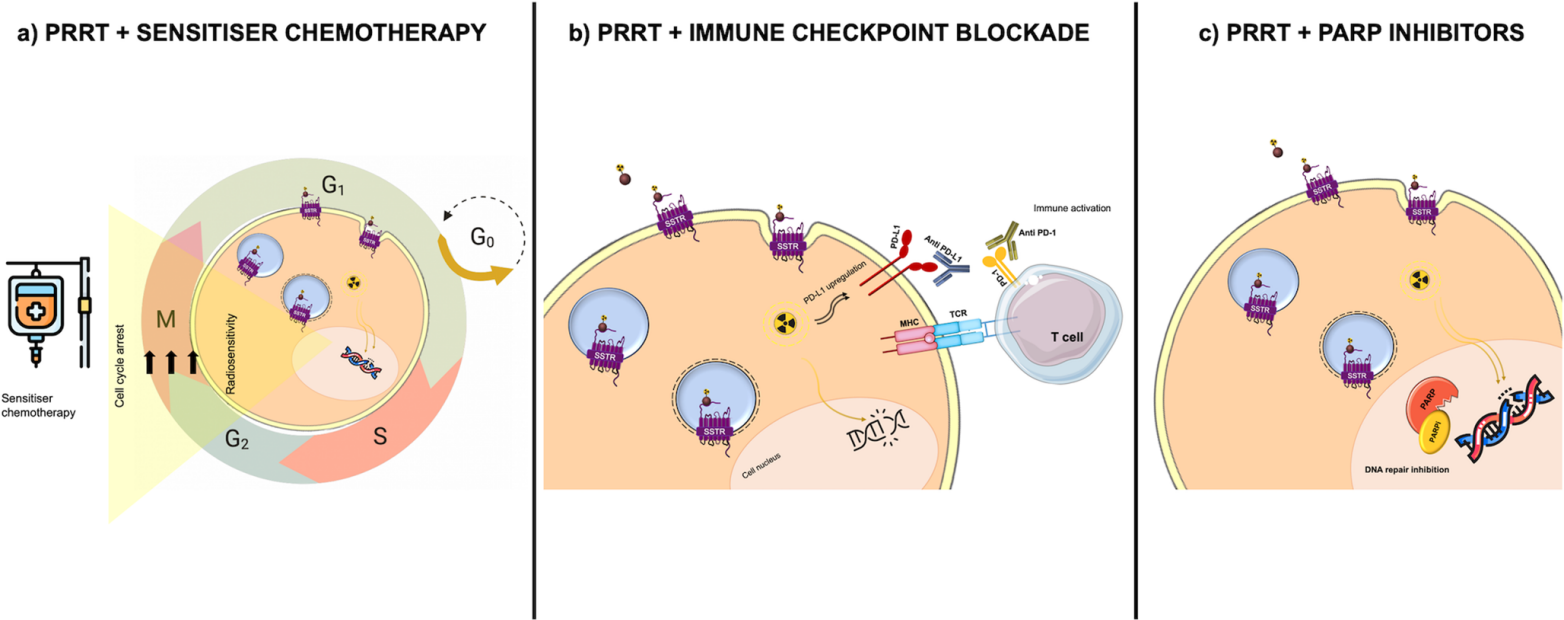
Mechanisms and combination strategies to enhance the efficacy of Peptide Receptor Radionuclide Therapy (PRRT). This illustration summarises key therapeutic mechanisms that can potentiate PRRT, including: (**a**) radiosensitisation through chemotherapy; (**b**) induction of immunogenic cell death, enabling synergistic combinations with immune checkpoint inhibitors; and (**c**) inhibition of DNA damage repair pathways (e.g., PARP inhibitors). These strategies may enhance DNA double-strand break induction, improve tumour response, and increase the therapeutic index

(Figure 2 and Table 2)

**Table 2:** Active recruiting and not recruiting clinical trials on combination strategies with PRRT (status December 2025)

Combination	No Trial	Phase	Patient	*N*	Centre/Sponsor
Chemoradionuclide Therapy					
PRRT + CAP	NCT05387603	III	Advanced/metastatic, progressive, well-differentiated, inoperable NETs of any primary origin and any grade, except for PPGL	300	Lund University Hospital
PRRT + CAPTEM	NCT07185672	III	Locally advanced/inoperable or metastatic G1-G3 GEP-NETs with disease progression in last 6 months	162	Tata Memorial Hospital
PRRT + Immune Checkpoint Inhibition					
PRRT + Avelumab	NCT04261855	Ib/II	Metastatic Merkel Cell Carcinoma	19	Melanoma and Skin Cancer Trials Limited
PRRT + Pembrolizumab	NCT05583708	II	Metastatic Merkel Cell Carcinoma	18	Weill Medical College of Cornell University
PRRT + Pembrolizumab	NCT03457948	II	Advanced/metastatic, progressive, well-differentiated (G1, G2, or G3) NETs of any primary site, including unknown primary	32	University of California, San Francisco
PRRT + PARP Inhibition					
PRRT + talazoparib	NCT05053854	I	G2 NETs (Ki-67 3–20%) from pancreatic or intestinal origin	24	Peter MacCallum Cancer Centre, Australia
PRRT + olaparib	NCT06607692	II	Children and adolescents with recurrent or relapsed solid tumours expressing SSTR	25	Fundación de investigación HM
PRRT + olaparib	NCT05870423	I	Locally advanced or metastatic, well-differentiated (G1, G2 or G3) NETs progressed following initial/salvage PRRT or with no suitable systemic alternative options	24	Erasmus Medical Center
PRRT + olaparib	NCT04086485	I/II	Advanced/metastatic, progressive, well-differentiated, inoperable GEP-NETs	56	National Cancer Institute
Other Combinations					
PRRT (neoadjuvant) + surgery	NCT04609592	I	Metastatic G1-G2 (Ki-67 ≤ 20%) GEP-NETs (with lymph nodes or liver metastases only), candidated for cytoreductive surgery	10	Stanford University
PRRT + SBRT	NCT07150546	I	Well-differentiated, G1-G2 GI NETs, unresectable, progressive after one or two prior lines of systemic therapy	15	Emory University
PRRT + Fulvestrant	NCT06663072	II	Advanced/metastatic, progressive, well-differentiated, G1-G2 (Ki-67 ≤ 20%) pNET	25	University of Chicago
PRRT + Sunitinib	NCT05687123	I	Metastatic, unresectable well- or moderately-differentiated pNETs of any grade	24	National Cancer Institute
PRRT + Triapine	NCT05724108	II	Metastatic, progressive, well-differentiated NETs exept for lung NETs	94	National Cancer Institute
PRRT + Triapine	NCT04234568	I	Metastatic, progressive, well-differentiated NETs	31	National Cancer Institute
PRRT + Peposertib	NCT04750954	I	Advanced/metastatic, progressive, well-differentiated, inoperable GEP-NETs	29	National Cancer Institute
PRRT + Cabozantinib	NCT05249114	I	Advanced/metastatic, progressive, well-differentiated (G1, G2, or G3) GEP-NETs, including unknown primary oring	6	Providence Health & Services
PRRT + Cedazuridine + Decitabine	NCT05178693	I	Advanced/metastatic, progressive, well-differentiated (Ki-67 < 55%) NETs	27	Imperial College London

Legend: *CAP *capecitabine, *GEP-NETs *gastroenteropancreatic neuroendocrine tumours, *NETs* neuroendocrine tumours, *TEM* temozolomide, *pNET * pancreatic neuroendocrine tumours, *PPGL *paraganglioma, *SBRT* stereotactic body radiation therapy, *SSTR *somatostatin receptors

### Mechanistic framework

Combination therapies with PRRT merge radiobiological and systemic mechanisms for improved therapeutic indices. Radiosensitisation and augmented treatment efficacy arise through [44]:

- enhanced DNA damage and impaired repair (e.g., PARPi, temozolomide).
- improved perfusion and oxygenation (anti-VEGF).
- SSTR up-regulation via certain cytotoxics.
- immune activation by neoantigen exposure.

### Chemoradionuclide therapy

The AGITG CONTROL NET study [45] randomised patients with pancreatic or midgut NETs to [^177^Lu]Lu-DOTATATE ± CAPTEM (i.e., capecitabine + temozolomide). Whereas no significance was found for midgut NETs, in p-NETs the median PFS in the combination group (PRRT + CAPTEM) was not reached vs. 14 months for CAPTEM alone, with objective response rate 44% vs. 28%, respectively, and an acceptable safety profile with manageable haematotoxicity.

A complementary Innsbruck trial using [^18^F]FDG PET/CT to guide PRRT + CAPTEM selection confirmed improved outcomes in metabolically active lesions, suggesting that [^18^F]FDG PET/CT may serve as a biomarker for radiosensitisation [46].

As reported above [38], [^225^Ac]Ac-DOTATATE and capecitabine in 91 patients showed promising results in both [^177^Lu]Lu-PRRT pretreated and [^177^Lu]Lu-PRRT-naive patients.

### PRRT + Immune checkpoint Inhibition

The rationale is based on the fact that PRRT-induced DNA damage promotes antigen release and PD-L1 up-regulation, potentially sensitising tumours to immunotherapy. Preclinical models suggest that T-cell infiltration is enhanced and improved survival may be achieved when PRRT precedes anti-PD-1 by several days [47].

First clinical evidence includes:

- [^**177**^**Lu]Lu-DOTATATE + nivolumab**: phase I trial (NCT04525638) demonstrated acceptable toxicity and 30% partial responses in lung NETs [48].
- **[**^**177**^**Lu]Lu-DOTATATE + pembrolizumab**: Pilot NANETS 2024 study [49]: ORR 34.6%, median PFS 11.2 months, with grade 3–4 toxicities < 10% in G2 and G3 advanced NETs.
- **[**^**177**^**Lu]Lu-DOTATATE + avelumab**: phase I/II trial (NCT04261855) in Merkel Cell Cancer is active and recruiting.

### PRRT + PARP Inhibition

The rationale is based on that DNA-repair inhibition amplifies PRRT-induced double-strand breaks. The LuPARP phase I trial [50] combined [^177^Lu]Lu-DOTATATE with olaparib (50–300 mg twice per day). The combination was feasible; thrombocytopenia was dose-limiting. The recommended phase II dose is 200 mg twice per day. Further trials aim to quantify synergistic efficacy (NCT05870423; NCT04375267; NCT05053854).

### Other combinations

- **PRRT + mTOR inhibitors**: Everolimus enhances radiosensitivity via PI3K-AKT pathway inhibition [51].
- **PRRT + anti-angiogenic therapy**: Bevacizumab normalises tumour vasculature, improving radiopharmaceutical distribution [44].
- **PRRT + [**^**131**^**I]I-MIBG**: Combined SSTR and norepinephrine transporter targeting achieved additive tumour-dose increases (34–83%) without additional toxicity [52].
- **PRRT (neoadjuvant) + surgery + PRRT after surgery**: in the phase II NEOLUPANET trial (NCT04385992) objective partial response was reached in 58% of non-functioning p-NETs with no progressive disease among the 31 patients treated with neoadjuvant PRRT [53]. Stanford University is currently running a phase I clinical trial (NCT04609592) evaluating neoadjuvant PRRT in patients with well-differentiated G1–G2 GEP-NETs who are candidates for cytoreductive surgery.
- **PRRT + stereotactic body radiation therapy (SBRT)**: NCT07150546.
- **PRRT combination with inhibitors of ribonucleotide reductase**, **tyrosine kinase**, **DNA-dependent protein kinase or DNMT inhibitors** (studies NCT04234568; NCT05724108; NCT04750954; NCT05687123; NCT05249114; NCT05178693).

## Novel peptide ligands and theragnostic advances

### Beyond somatostatin receptors

The success of PRRT has encouraged exploration of alternative receptors expressed in NETs and other malignancies, including CCK2R, GRPR, and GLP-1R. Each offers unique opportunities for imaging and therapy in tumours lacking SSTR2 expression.

### Cholecystokinin-2 receptor (CCK2R) targeting

The CCK2R is over-expressed in MTC (~ 90%), SCLC (~ 57%), other GEP-NETs (~ 22%) and may also be over-expressed in certain sarcoma types [54].

#### Radioligand development

- **First-generation compounds**: [^111^In]In-DTPA-MG0 (“Marburg compound”) and [^90^Y]Y-DOTA-MG11 showed proof-of-principle but suffered from metabolic instability and high kidney uptake [55–57].
- **Next-generation analogues**: [^177^Lu]Lu-PP-F11N and [^111^In]In-CP04 (DOTA-DGlu₆-Ala-Tyr-Gly-Trp-Nle-Asp-Phe-NH₂) displayed improved stability and favourable tumour-to-kidney ratios [58].
- **DOTA-MGS5**: Developed at Innsbruck, this ligand incorporates N-methylation and modified DGlu linkers for enhanced in vivo stability and reduced renal retention [59].

#### Preclinical and clinical data

Mouse models demonstrated high tumour uptake and 10-fold improved tumour-to-kidney ratios [59]. A first-in-human study (NCT06155994) confirmed safety and favourable biodistribution in 12 patients (6 MTC, 6 other NETs) [17].

The ongoing multicentre phase II program will define therapeutic efficacy of [^177^Lu]Lu-DOTA-MGS5 in patients with SCLC (EUCT ID 2024–514584-25-00; 2024–518039-12-00). The compound [^177^Lu]Lu-PP-F11N is applied in patients with MTC (NCT03647657; [60]).

### Other emerging ligands

Beyond somatostatin receptor–directed approaches, several additional peptide receptor systems are being explored to broaden theragnostic options, particularly in NETs or neuroendocrine-like tumours with heterogeneous or low SSTR expression.

#### GRPR-targeted radioligands

Gastrin-releasing peptide receptor (GRPR) antagonists (e.g., [^68^Ga]Ga-RM2, [^177^Lu]Lu-NeoBOMB1) have shown promising diagnostic and therapeutic properties in solid tumours with high GRPR expression, including prostate cancer, breast cancer, and gastrointestinal stromal tumours (GIST). The MITIGATE-NeoBOMB1 first-in-human phase I/IIa study demonstrated favourable safety, pharmacokinetics, and imaging performance of [^68^Ga]Ga-NeoBOMB1 in GIST patients [61]. More recently, Baratto et al. from Stanford reported encouraging early-phase results for GRPR-targeted PET imaging in solid tumours, further supporting the clinical potential of this theragnostic class [62, 63]. The NeoRay phase I/IIa trial (NCT03872778), now closed to recruitment, further investigates the safety, biodistribution and preliminary antitumour activity of [^177^Lu]Lu-NeoB in GRPR-positive solid tumours.

#### GLP-1 receptor ligands for insulinoma

Insulinomas frequently show low SSTR expression, limiting the sensitivity of conventional SSTR-PET. In this setting, [^68^Ga]Ga-Exendin-4, a GLP-1 receptor agonist [64], has proven highly effective for localising benign and malignant insulinomas, as highlighted by Christ et al. [65]. At present, GLP-1R targeting is essentially synonymous with [^68^Ga]Ga-Exendin-4, as no other clinically validated GLP-1R radiotracers are widely used.

#### Dual-receptor targeting

Dual-receptor approaches aim to overcome intratumoral heterogeneity by combining two peptide tracers or ligands targeting different receptor systems.

Reubi and Waser [54] demonstrated the biological rationale for dual targeting through systematic receptor autoradiography, providing evidence that combined SSTR and GRPR (or CCK2R) targeting may substantially enhance tumour visualisation and therapeutic applicability.

Such strategies may be especially relevant for tumours with mixed receptor expression patterns or partial SSTR negativity and illustrate the rapid expansion of multi-receptor theragnostics in personalised nuclear oncology.

## Dosimetry, safety, and long-term outcomes

Accurate dosimetry is essential for optimising the therapeutic window of PRRT, ensuring that tumour irradiation is maximised while protecting organs at risk. Individualised dosimetry with SPECT/CT enables reliable estimation of organ-specific absorbed doses and supports tailoring the number and activity of treatment cycles to the patient’s physiology.

Key organ dose thresholds generally accepted in clinical practice include [66]:

- **Kidneys**: cumulative absorbed dose below approximately 23 Gy to minimise long-term nephrotoxicity.
- **Bone marrow**: less than 2 Gy per treatment cycle to limit significant haematological toxicity.
- **Secondary malignancies**: the rate of treatment-related MDS/AML remains low (< 1%) even after follow-up periods exceeding eight years.

A detailed dosimetric analysis conducted within the NETTER-1 study [67] revealed substantial inter-individual variability in the absorbed tumour dose. Among patients treated with four standard cycles of 7.4 GBq [^177^Lu]Lu-DOTATATE, the median tumour dose was approximately 134 Gy, corresponding to an estimated 50% likelihood of achieving a partial tumour response. Additional investigations confirm a clear dose–response relationship in NETs treated with [^177^Lu]Lu-DOTATATE, with higher absorbed tumour doses associated with improved objective response rates and prolonged disease control [68].

Furthermore, re-challenge with PRRT in patients who previously achieved clinical benefit has demonstrated disease control rates exceeding 60%, while preserving renal function, underscoring both the safety and therapeutic relevance of PRRT retreatment [69, 70].

However, long-term safety considerations remain critical. Although the overall incidence of late adverse events such as MDS or AML is low (< 1%), careful patient selection, cumulative dose monitoring, and lifelong follow-up are essential to detect potential delayed toxicities, particularly in heavily pretreated individuals or those receiving combination or sequential therapy.

## Discussion

During the past decade, PRRT has evolved from a niche salvage treatment into a mainstream modality within the multimodal management of NETs. Evidence from randomised and prospective studies demonstrates that PRRT offers durable tumour control, symptomatic relief, and improved quality of life with a low incidence of severe toxicity. The success of [^177^Lu]Lu-DOTATATE has stimulated an unprecedented expansion of the field in both clinical and translational dimensions.

### Integration into treatment algorithms

The findings of NETTER-1 and NETTER-2 have established PRRT not only as standard second-line therapy but also as a candidate for first-line use in SSTR-positive, intermediate-grade NETs [4, 5, 12]. Increasingly, multidisciplinary tumour boards are considering PRRT earlier in the disease course, especially for patients with high SSTR expression, limited tumour burden, and favourable performance status. Dosimetry-guided therapy is progressively replacing fixed-dose schedules, enabling personalisation of activity per cycle and cumulative dose limits [71].

### Personalised and Biomarker-Driven approaches

The integration of molecular imaging biomarkers, such as combined [^68^Ga]Ga-SSTR PET/CT and [^18^F]FDG PET/CT, refines patient selection by capturing tumour heterogeneity. High [^18^F]FDG uptake predicts aggressive biology and may indicate benefit from treatment combinations (e.g., CAPTEM + PRRT) [46]. Emerging biomarkers—including circulating miRNAs, gene signatures, and DNA-damage response proteins—may in the future guide the sequencing of PRRT, α-therapy, and systemic agents.

### The promise of α-emitters

Targeted α-therapy represents the most potent innovation in radioligand therapy. Early experiences with [^225^Ac]Ac-DOTATATE and ^212^Pb-labelled compounds reveal durable responses even in β-refractory patients, with acceptable toxicity. However, widespread adoption requires progress in radionuclide supply, automated labelling chemistry, and dosimetric modelling of daughter nuclide recoil. Results from ongoing clinical trials could pave the way for a better understanding of α-PRRT, helping to clarify the efficacy and safety profiles of the various compounds under investigation [36]. The development of hybrid α/β treatment algorithms—alternating or sequential application—may achieve optimal tumour control while preserving organs at risk [72].

### Combination and sequential concepts

Combination strategies with chemotherapy, DNA-repair inhibitors, or immune checkpoint blockade exploit distinct biological mechanisms:

1. increasing DNA damage beyond repair;
2. modulating tumour perfusion and hypoxia; and
3. inducing immunogenic cell death.

The encouraging early results of CAPTEM + PRRT and LuPARP trials justify further randomised evaluation. Particular attention should be given to sequencing and timing, as pre-clinical models suggest maximal synergy when PRRT precedes immunotherapy by several days [47]. Sequential regimens integrating PRRT with everolimus, sunitinib, or bevacizumab also merit exploration.

### Expanding the receptor landscape

The identification of alternative peptide receptors such as CCK2R, GRPR, and GLP-1R has broadened theragnostic horizons beyond SSTR. The Innsbruck-developed DOTA-MGS5 exemplifies this evolution—combining high receptor affinity, metabolic stability, and favourable dosimetry [18, 59]. First-in-human data confirm safety and feasibility in MTC and SCLC, supporting further multicentre phase II studies. In parallel, dual-receptor imaging and therapy may overcome inter- and intra-tumoural heterogeneity.

### Regulatory and logistical considerations

As new isotopes and ligands enter the clinic, harmonised production standards and dosimetric reporting are essential. Regulatory frameworks must adapt to allow early access under compassionate-use or named-patient programmes while safeguarding safety monitoring. Collaborative networks such as ENETS, WARMTH and the IAEA play a crucial role in coordinating multicentre trials and fostering translational collaboration between nuclear medicine, oncology, and radiopharmacy across Europe as well as the globe [73].

## Future perspectives 

(Figure 3)

**Fig. 3:**
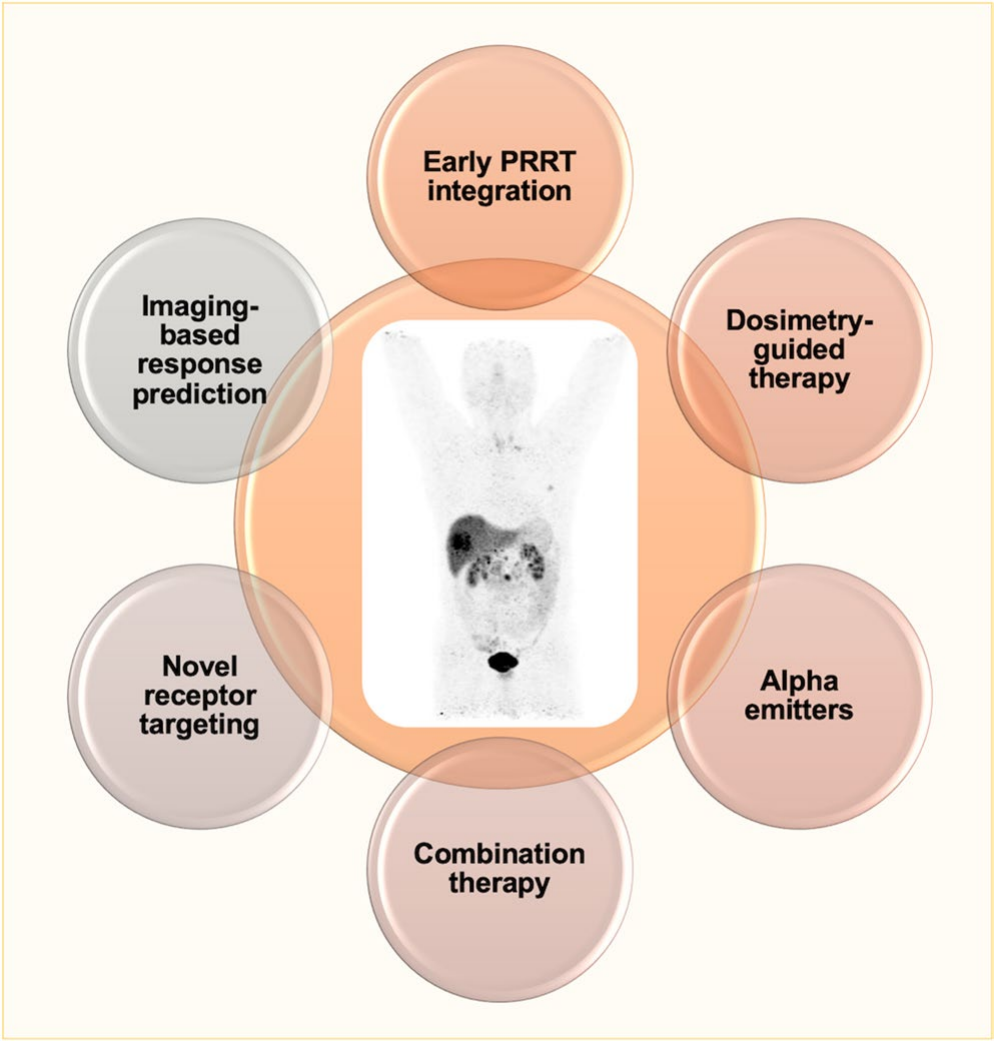
Peptide receptor radioligand therapy (PRRT) pathway from diagnosis to personalized therapy. Schematic overview of the patient journey in contemporary PRRT, highlighting key elements of future PRRT development. These include baseline dosimetry assessment, earlier integration of PRRT into the treatment pathway, optional escalation to α-emitter therapy or novel receptor targeting strategies, and biomarker- and imaging-guided treatment intensification with response prediction

PRRT exemplifies the shift from organ-based to molecularly-defined oncology. Future directions can be summarised as follows:

1. **Early-line integration and adjuvant concepts**: evaluating PRRT in neoadjuvant or consolidation settings after surgery or systemic therapy.
2. **Adaptive**,** dosimetry-guided therapy**: employing SPECT/CT-based dose monitoring to individualise cycles and maximise therapeutic index.
3. **α-emitter expansion**: defining optimal radionuclide-ligand combinations ([^225^Ac]Ac-DOTA-MGS5, [^212^Pb]Pb-DOTAMTATE) for resistant disease.
4. **Multimodal combination therapy**: rational design of trials combining PRRT with chemotherapy, immunotherapy, PARP inhibition, or anti-angiogenic agents.
5. **Novel receptor targeting**: translation of CCK2R and GRPR ligands to phase III development for rare NETs and extrapulmonary neuroendocrine carcinomas.
6. **Imaging-based response prediction**: incorporation of radiomics, artificial intelligence, and immune-checkpoint PET (e.g., [^89^Zr]Zr-atezolizumab) for precision selection.

Together, these directions envision PRRT as the backbone of a comprehensive theragnostic platform capable of addressing tumour heterogeneity, resistance, and individual patient biology.

## Conclusion

Peptide receptor radionuclide therapy has transformed the management of neuroendocrine tumours and continues to expand its reach through innovation in radiochemistry, physics, and molecular biology. The convergence of β- and α-emitter technologies, antagonist ligands, and synergistic systemic combinations marks the beginning of a new era in precision nuclear oncology. Sustained international collaboration and harmonised clinical trial networks will be crucial to translate these advances into improved survival and quality of life for patients with neuroendocrine neoplasia.

## Data Availability

Not applicable. All data used for this review can be retrieved from the reference list.
